# A Case of Accidental Isoniazid Overdose Presenting With Nonspecific Symptoms

**DOI:** 10.7759/cureus.23218

**Published:** 2022-03-16

**Authors:** Edgar Asiimwe, Michelle Koh, Rajan Patel

**Affiliations:** 1 Internal Medicine, University of California Los Angeles, Los Angeles, USA; 2 Internal Medicine, Olive View - UCLA (University of California Los Angeles) Medical Center, Los Angeles, USA

**Keywords:** latent tuberculosis, overdose, hepatology, acute liver failure, inh, isoniazid, tb

## Abstract

A 68-year-old male with a history of end-stage renal disease and latent tuberculosis on isoniazid (INH), and no psychiatric history presented with a five-day history of anorexia, fatigue, and nausea. Physical exam in the emergency department was notable for somnolence, right upper extremity tremor, and diffuse abdominal pain. Initial workup revealed an anion gap metabolic acidosis with elevated lactate, prompting admission to the general ward for empiric IV antibiotics for suspected bacteremia from his permacath.

Within a few hours of admission, he became increasingly encephalopathic and had two episodes of copious hematemesis. Repeat studies revealed a cholestatic pattern of liver injury and new-onset coagulopathy. With an overall clinical picture consistent with fulminant hepatic failure, our pharmacy team initiated a comprehensive pill count of all his medications, which established that he had been inadvertently taking up to six times the recommended dose of INH.

With INH discontinuation and supportive therapy, he improved and was discharged on hospital day eight. Our experience provides lessons in the timely recognition and management of this rarely reported toxidrome in the United States.

## Introduction

Isoniazid (INH) is the first-line therapy for latent tuberculosis (TB) either as monotherapy or in combination with other agents at many centers [1]. Typical monotherapy dosing is at 5 mg/kg with a daily maximum recommended dose of 300 mg for nine months; doses above 300 mg are associated with increased risk of hepatotoxicity and neurotoxicity, with acute INH toxicity observed at ingestions in excess of 2g [1].

But even at the recommended 300 mg daily dosing, INH carries a significant risk of hepatotoxicity and neurotoxicity [2,3]. This risk increases with age and other extrinsic variables such as concomitant use of other hepatotoxic medications and alcohol use. For this reason, at initiation of therapy, at-risk patients (defined as those over the age of 35 or those with comorbidities) are monitored monthly for adverse reactions with monthly liver function tests (LFTs) for the first three months of therapy, a period during which the risk of hepatotoxicity is highest [2,3]. In addition, all patients are prophylactically prescribed pyridoxine to decrease the risk of INH-induced toxicity [2].

INH toxicity, in-vitro studies have demonstrated, results from the accumulation of toxic hydrazine metabolites generated as the drug is metabolized: INH is first acetylated to its active metabolite acetyl-INH (Ac-INH) in the liver and then hydrolyzed to its hydrazine form [3,4]. These hydrazine metabolites then undergo further acetylation to inactive but toxic metabolites which are excreted in urine. The products of this second acetylation step can be toxic and exert their deleterious effects in three main ways: (1) They impair mitochondrial function leading to accumulation of toxic reactive oxygen species and ultimately hepatocyte death [3,5]; (2) They lead to decreased pyridoxine levels, achieved by inhibition of pyridoxine phosphatase (the enzyme needed to convert pyridoxine to its 5’-phosphate active form) and by active consumption of pyridoxine itself during the acetylation step [3,5]; (3) They lead to impaired gamma-aminobutyric acid (GABA) synthesis-achieved via the depletion of pyridoxine, an important co-enzyme for glutamate decarboxylase (GAD) activity [3,5,6].

The classic signs and lab findings of acute INH toxicity align with the drug’s metabolism described above: inadequate GABA synthesis increases the risk of encephalopathy and seizures, while mitochondrial impairment leads to transaminitis, elevated lactate (due to impaired aerobic respiration), and hepatocyte death in turn precipitating life-threatening liver failure in severe cases [5-8].

While acute INH overdose can be life-threatening, early recognition and treatment portend a good prognosis [9-11]. Timely recognition requires high clinical suspicion, which can be clouded by both the nondescript symptomatology of the toxidrome shared with myriad other conditions and the relatively low prevalence of INH toxicity in the United States: from 2009 to 2014, there were 1373 cases of INH overdose cases reported to the American Association of Poison Control Centers’ National Data Collection System, representing 0.0013% of all reported toxic ingestions within that time frame [12]. Moreover, to further underscore the toxidrome’s rarity, the historically-recommended antidote (intravenous (IV) pyridoxine) is rarely in-stock at hospital pharmacies: in one case report, the authors narrate how their search for a 9 g dose of pyridoxine, needed to treat one patient, all but depleted an entire state’s supply of the drug [13].

With this report, we share our experience about a patient who presented with nausea, abdominal pain, an anion gap metabolic acidosis, and a cholestatic pattern of hepatic injury in the setting of inadvertent INH overdose. This case occurs at a safety-net hospital in the United States (US) serving a large immigrant population from TB-endemic regions. Given the toxidrome’s extremely low prevalence, overlap of its nondescript (at least in the early stages) signs and symptoms with other etiologies, and high risk of fatal complications, our experience serves as a reminder to providers, particularly those serving a catchment population similar to ours, to consider this etiology.

## Case presentation

The patient presented to our emergency department (ED) endorsing fatigue, malaise, anorexia, nausea, and hyper-somnolence. Pertaining to symptom chronicity and order, he stated that the generalized fatigue had appeared first (five days prior to presentation). At that time, he had initially attributed his symptoms to the COVID-19 vaccine, having received his second dose around the same time as symptom onset. As a result, he had not sought medical attention, thinking the symptoms would self-resolve.

However, the fatigue progressed, and two days prior to presenting, he developed hyper-somnolence, diaphoresis, loss of appetite, and nausea without emesis. This constellation of symptoms was debilitating enough that he missed a scheduled hemodialysis session (HD), opting instead to present to the ED.

In the ED, a review of systems was negative for localizing symptoms of infection such as cough, rhinorrhea, and or diarrhea. His past medical history was notable for end-stage renal disease requiring three-times-a-week dialysis, latent TB, and type II diabetes. When asked about his home medications, he provided a bag with pill bottles, stating that he had been taking all medications as instructed on the bottle. His medications were: isoniazid (300 mg daily, first prescribed four months prior to admission), amlodipine, aspirin, ergocalciferol, ferrous sulfate, lisinopril, folic acid, furosemide, multivitamin, sevelamer, and pravastatin.

In terms of social history, he denied any alcohol or illicit drug use and denied any recent sick contacts. He worked as a school bus driver, was born in Mexico, but had been living in the US for the past 20 years without any recent travel.

On exam, his vitals were within normal limits. However, on a neurologic exam, he was somnolent but arousable and oriented to person, place, and time. A right-sided upper extremity intention tremor was also noted. On abdominal exam, diffuse abdominal tenderness without guarding or rebound was noted. On visual inspection, a right internal jugular permacath for HD with a clean dressing was noted; there was no overlying erythema, tenderness, or purulence. No other potential ports for infection were noted, and the rest of the exam was unremarkable.

Within a couple of hours, the patient became increasingly altered and delirious and began pulling at his IV lines. Bedside exam revealed stable vitals, but with a neurologic exam notable for worsening orientation and audio-visual hallucinations. As a result, a 1:1 sitter was placed for safety. Within a few hours, he had two episodes of copious hematemesis prompting emergent consultation of our intensive care unit (ICU) service and transfer to our step-down unit for more frequent monitoring and in the case that emergent intubation would be needed.

This rapid decline in clinical status was concerning for acute liver injury. As a result, to elucidate etiology, a thorough pharmacy review was initiated, and an in-house technician contacted the patient’s wife in order to perform a full pill count. The audit revealed that the patient had been taking six times the prescribed dose of INH. As it were, two weeks prior, the patient had been asked, by his HD physician, to double his sevelamer dose from 800 mg three times a day to 1600 mg three times a day. However, the patient and his wife had mistakenly applied those instructions to his INH dose. With this additional information, INH was indefinitely held. We also discontinued his pravastatin due to its hepatotoxic side effect profile.

Diagnostics

Initial workup included a comprehensive metabolic panel (CMP) notable for an anion-gap metabolic acidosis in the setting of both lactic acidosis and azotemia (Table 1). A complete blood count (CBC) was entirely within normal limits without either leukocytosis or leukopenia. A hepatic panel was notable for transaminitis with an R-Factor of 0.3 on admission suggesting a cholestatic pattern of liver injury at the outset. Later, the R-factor increased to 2.1, suggesting mixed hepatic injury (Table 1).

**Table 1:** Comprehensive metabolic panel Values outside normal ranges ascribed H-(high); L-(low)

Laboratory parameter	Value
Sodium	139 mmol/L
Potassium	(H) 5.2 mmol/L
Chloride	100 mmol/L
Bicarbonate	(L) 19 mmol/L
Anion gap	(H) 20 mmol/L
Urea	(H) 83 mg/dL
Creatinine	(H) 11.24 mg/dL
Glucose	130 mg/dL
Calcium	(L) 7.7 mg/dL
Phosphorus	7.9 mg/dL
Albumin	(L) 2.7 g/dL
Alkaline Phosphatase (ALP)	(H) 137 U/L
Aspartate Aminotransferase (AST)	(H) 426 U/L
Alanine Transferase (ALT)	37 U/L
Total Bilirubin	(H) 2.3 mg/dL
Bilirubin, Direct	(H) 1.5 mg/dL
Ammonia	30 (<45)

Given the transaminitis and abdominal pain, a non-contrast CT of the abdomen and pelvis was obtained revealing diffuse hepatic hypoattenuation concerning for hepatic steatosis, but no signs of cirrhosis (not shown). The rest of the study was unrevealing. An acute hepatitis panel was notable for positive anti-smooth muscle antibodies (Table 2), but the patient did not meet objective criteria for autoimmune hepatitis.

**Table 2:** Additional biochemical studies Values outside normal ranges ascribed H-(high); L-(low)

Laboratory parameter	Value
Creatinine Kinase	74 (N)
Hepatitis A Immunoglobulin (Ig)M	Negative
Acute Hepatitis B serologies	Negative
Hepatitis C IgG	Negative
Antinuclear antibodies (ANA)	Negative
Anti-smooth Muscle Antibody	Positive; 1:40
Anti-Liver Kidney Microsome Type I (LKM-1) antibody	Negative
Lactate	(H) 4 mmol/L (normal <0.5)

When the patient’s mental status deteriorated later on day one of hospitalization, we obtained a non-contrast CT of the brain, which was unremarkable (not shown).

Treatment

Prior to transfer to the medicine floor, the patient had been started on empiric antibiotics (vancomycin/piperacillin-tazobactam) owing to initial suspicion for bacteremia with his indwelling permacath as a suspected source. Both peripheral and line blood cultures had been sent prior to antimicrobial initiation. All cultures remained negative and antibiotics were stopped after 48 hours.

When the patient developed hematemesis, IV pantoprazole, desmopressin, and fresh-frozen-plasma (for an international normalized ratio (INR) of 4; normal <1), and vitamin K were administered. In addition, the ICU team was emergently consulted for intubation, but the hematemesis had resolved by the time of evaluation. Nonetheless, the patient was immediately transferred to our step-down unit for closer monitoring and ease of intubation in case the hematemesis were to re-emerge.

Given concern for acute liver failure from drug-induced liver injury (DILI), the transfer process to a regional transplant center was initiated. For the patient’s encephalopathy, characterized by hyperactive delirium with altered sleep-wake cycle, we maintained a 1:1 sitter for frequent re-orientation and monitoring and initiated lactulose titrated to three bowel movements per day.

Outcome

By end of day one of hospitalization, no further hematemesis had been observed and, as a result, the patient was not intubated. By day three, the patient’s mental status had begun to improve with a return to baseline mental status by day four. Physical therapy to prevent de-conditioning was initiated at that point.

**Figure 1:**
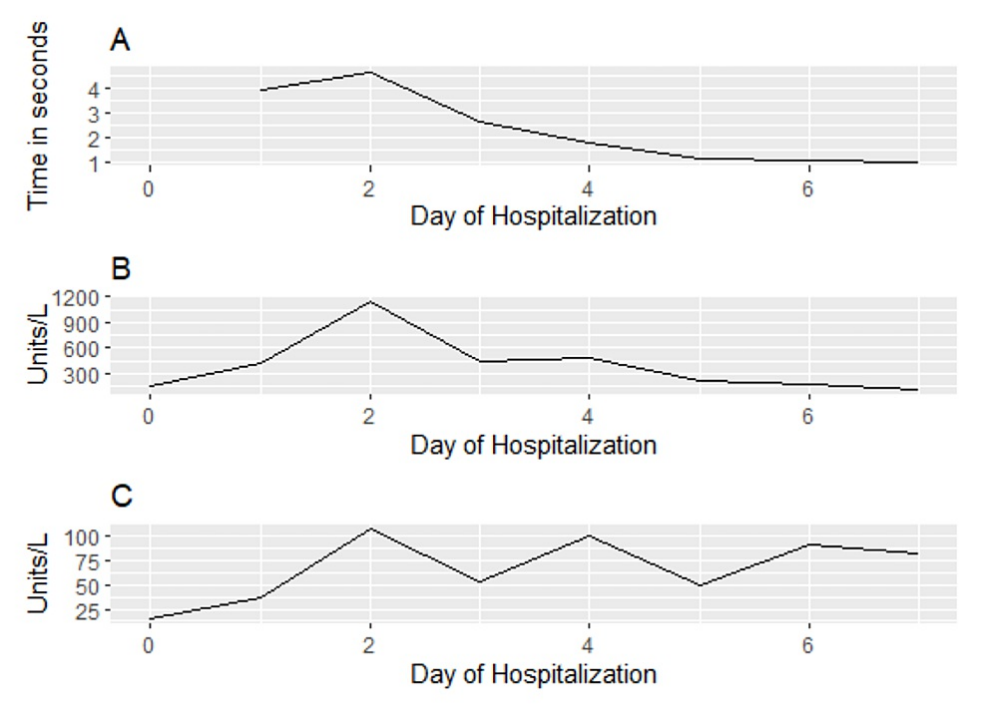
Trend in key hepatic biomarkers over hospitalization course A: international normalized ratio (INR), B: aspartate aminotransferase, C: alanine aminotransferase

The patient was discharged on day eight with a normalized INR of 1.08 (from a peak of 4), baseline mental status, and improving LFTs (Figure 1). INH was not restarted at discharge, and the patient was urged to follow up with his outpatient physician.

## Discussion

In the past decade, reports of acute INH toxicity in the US have been comparatively sparse, with most cases occurring in the setting of intentional overdose [12]. As a result, except among patients with suspected suicidal intent, this diagnosis likely has a low pretest probability among clinicians. However, as our experience demonstrates, timely recognition is crucial owing to the significant risk of rapid clinical deterioration primarily from liver failure.

Regarding treatment, we observe supportive management as crucial to eventual positive outcomes in this cohort: our experience notably differs from that in most of the published studies in that we did not administer IV pyridoxine as part of our treatment strategy which, in addition to supportive care, is the oft-cited treatment of choice in the literature. Pyridoxine's potential as an antidote for INH overdose was first demonstrated in a 1981 case series of five patients in which the authors empirically administered IV pyridoxine equivalent to the ingested amount of INH with positive outcomes [9]. But that study had a number of limitations, a small sample size notwithstanding, including the use of historical controls as the comparative group and internal validity concerns as the effect of additional interventions administered to patients was not accounted for. As a result, there is no robust empiric evidence of the efficacy of this intervention. Nonetheless, our experience identifies supportive care as a potent component of management.

We also noted unusual laboratory findings, including a cholestatic pattern of liver injury with aspartate aminotransferase (AST) dominance (as opposed to the hepatocellular pattern of injury typically seen with INH toxicity), and a positive anti-smooth muscle titer. The cause for the elevated AST, we postulate, is the fact that the patient presented during the early phase of hepatic injury during which cellular damage is localized to the mitochondria; during this phase, the cell’s cytosol is able to ward off global apoptosis by translocating inhibitors from the mitochondria to the cytosol, a process achieved by increasing mitochondrial membrane permeability [14,15]. This increased permeability likely facilitates AST diffusion leading to the observed increase in serum levels. Subsequently, in the later stages of damage, ALT levels rise when this protective mechanism becomes overwhelmed and global apoptosis ensues.

The positive anti-smooth muscle titer, we hypothesize, was likely a false positive as the patient did not meet the criteria for autoimmune hepatitis; furthermore, with a Naranjo score of six, the culpability of INH toxicity as the underlying etiology, in this case, is solidified.

Finally, while a root-cause analysis is beyond this paper’s scope, the role of social determinants of health deserves mention. Our facility serves a vulnerable population with limited medical literacy, a factor, in addition to miscommunication, that played a significant role in this outcome. Addressing negative upstream factors, we incidentally note, should remain a key consideration in the quest for health equity and improved outcomes.

## Conclusions

INH toxicity is a rarely reported cause of overdose (accidental or intentional) in the US, presenting with a nonspecific toxidrome that overlaps with other more prevalent etiologies. As a result, the condition may evade timely recognition with dire outcomes. This case provides diagnostic clues for its consideration, especially in the appropriate clinical and demographic setting. Furthermore, our experience highlights the critical importance of supportive care and aggressive management of liver failure in facilitating recovery.
